# COVID-19 vaccination intent and associated factors in healthcare workers: cross-sectional study: Ana Zhelyazkova

**DOI:** 10.1093/eurpub/ckac131.050

**Published:** 2022-10-25

**Authors:** A Zhelyazkova, S Kim, M Klein, S Prueckner, S Horster, P Kressirer, A Choukér, M Coenen, K Adorjan

**Affiliations:** 1 Institute of Emergency Medicine, University Hospital, LMU Munich, Munich, Germany; 2 Department of Neurology, University Hospital, LMU Munich, Munich, Germany; 3 Administrative Department, University Hospital, LMU Munich, Munich, Germany; 4 Department of Communication and Media, University Hospital, LMU Munich, Munich, Germany; 5 Laboratory of Translational Research Stress, University Hospital, LMU Munich, Munich, Germany; 6 Chair of Public Health, LMU Munich, Munich, Germany; 7 Pettenkofer School of Public Health, Munich, Germany; 8 Department of Psychiatry and Psychotherapy, University Hospital, LMU Munich, Munich, Germany

## Abstract

**Background:**

Considering the vaccine hesitancy recorded among healthcare workers (HCW) during the 2009/10 influenza pandemic, we aimed to examine the COVID-19 vaccination intent of HCW at one of the largest hospitals in Germany and to identify associated factors.

**Methods:**

We conducted a cross-sectional anonymous survey at LMU University Hospital in Munich, Germany, between February 25 and March 20, 2021. Data was collected on COVID-19 vaccination intent as main outcome and on potential associated factors.

**Results:**

In total, 2555 HCW completed the survey; 48,3% (n = 1325) of them had already received at least one COVID-19 vaccine dose. Of those not yet vaccinated 51,7% (n = 1320), 83,6% (n = 1104) reported intention to get vaccinated, while 10,2% (n = 134) were undecided and further 6,2% (n = 82) reported refusal. Disagreeing that everyone should receive the generally recommended vaccines was associated with refusal (RR = 529,500, p = 0,000) while being vaccinated against influenza in the 2020/21 season was linked with lower likelihood of refusal or indecisiveness (RR = 0,124, p = 0,000; RR = 0,182, p = 0,000). Low or partial conviction of the effectiveness and safety of COVID-19 vaccines were linked to refusal (effectiveness; RR = 485,471, p = 0,000; RR = 9,247, p = 0,000; safety: RR = 116,829, p = 0,000; RR = 5,423, p = 0,025). Feeling ill informed about COVID-19 vaccines was associated with refusal and indecisiveness (RR = 25,900, p = 0,000; RR = 21,104, p = 0,000).

**Conclusions:**

At the beginning of the vaccination campaign in Germany, a small proportion of HCW at LMU University hospital was hesitant on receiving a COVID-19 vaccine. Factors associated with refusal or indecisiveness were a sceptical attitude towards vaccines in general as well as feeling ill informed about COVID-19 vaccines, especially regarding their effectiveness and safety. Having received an influenza vaccine was associated with COVID-19 vaccination intent.

**Key messages:**

• The presented results provide insight into the reasons for hesitancy of HCW against COVID-19 vaccines, indicating a pattern-like behaviour in the acceptance of novel vaccines by HCW.

• The evidence from our analysis can help inform the communication aims and emphases of vaccination campaigns among HCW within similar organizational contexts or in future outbreak scenarios.

